# Isolated perinephric abscess as the initial manifestation of filariasis—an unusual presentation

**DOI:** 10.1002/ccr3.8608

**Published:** 2024-03-04

**Authors:** Govind Sharma, Rajat Sachdeva, Shweta Aggarwal, Amogh Verma, Ayush Anand, Ajeet Singh, Prakasini Satapathy

**Affiliations:** ^1^ Department of Radiodiagnosis Rama Medical College Hospital and Research Centre Hapur India; ^2^ Rama Medical College Hospital and Research Centre Hapur India; ^3^ BP Koirala Institute of Health Sciences Dharan Nepal; ^4^ Dow University of Health Sciences Karachi Pakistan; ^5^ Center for Global Health Research, Saveetha Medical College and Hospital, Saveetha Institute of Medical and Technical Sciences Saveetha University Chennai India; ^6^ Medical Laboratories Techniques Department AL‐Mustaqbal University Hillah Babil Iraq

**Keywords:** case report, filariasis, perinephric abscess

## Abstract

**Key Clinical Message:**

Filariasis may present as an isolated perinephric abscess. Hence, a high index of suspicion should be maintained in endemic settings.

**Abstract:**

In cases with unexplained fever, eosinophilia and perinephric collection, it is necessary to do detailed infectious disease work up. High index of suspicion is required to diagnose filariasis due to its wide range of clinical presentation and laboratory findings. It may present as perinephric abscess, which can be diagnosed through ultrasonography.

## Graphical abstract

Graphical Abstract

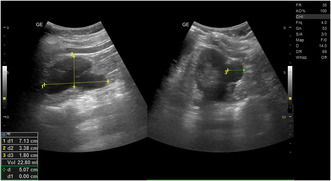

## 1 INTRODUCTION

Filariasis is an endemic disease in tropical countries of Africa, Southern America, and Asia where it is regarded as major public health problem. It is caused by parasitic roundworms that dwell in the blood and tissues of humans. *Wuchereria bancrofti* (90% of infections), and *Brugia timori* and *Brugia malayi* (10% of infections) are the most common organisms causing filariasis in humans.^1^ Of these, only *W. bancrofti* and *B. malayi* are found in India with *W. bancrofti* accounting for 98% of filarial infections in India.^2^ These nematodes are transmitted by mosquitoes. The mosquito vectors for filariasis vary geographically including the genus Culex, Anopheles, Aedes, Mansonia, and Coquillettidia. Humans are the so‐called definitive host where the sexual stages develop. A mosquito ingests the microfilariae again during a blood meal; these develop into larvae, which can infect another human when the mosquito takes a subsequent blood meal, completing the lifecycle.^3^ Lymphatic filariasis infection involves asymptomatic, acute and chronic conditions. The majority of infections are asymptomatic, showing no external signs of infection while contributing the transmission of the parasite. These asymptomatic infections still cause damage to the lymphatic system and the kidneys and alter the body's immune system. When lymphatic filariasis develops into chronic conditions it leads to lymphedema (tissue swelling) or elephantiasis (skin/tissue thickening) of limbs and hydrocele (scrotal swelling). Acute episodes of local inflammation involving skin, lymph nodes and lymphatic vessels often accompany chronic lymphedema or elephantiasis.^4^ There have been previous instances of finding filariasis at unique locations, where it is generally not found. Case reports have previously been published reporting its unusual sites such as breast, thyroid, body fluids, and skin. Oral or perioral involvement is even rarer.^2^ To the best of our knowledge, unilateral perinephric collection of filariasis is an extremely unique finding that has not been reported before in the global literature. Uniqueness of the case prompted us to report it.

## 2 CASE HISTORY/EXAMINATION

A 32‐year‐old married woman, presented to the outpatient department with complaints of low‐grade intermittent fever of unknown origin for the past 2 years. The fever ranged between 99.5°F (37.5°C) to 101.3°F (38.5°C), the patient had been managing the fever with regular paracetamol use, which temporarily alleviated symptoms for a few hours. Notably, during this extended duration, the fever was previously stable without significant exacerbations or changes in its pattern. Rest of the history was not found clinically significant. Following initial history taking, detailed physical examination was carried out, revealing mild tenderness in left lumber region, the patient reported that this symptom had been present intermittently for the past few months. However, there was a recent worsening of both fever and tenderness over the last 3 months, prompting the patient's visit to the outpatient department. The exacerbation of symptoms, particularly the tenderness, was concerning to the patient, as it deviated from the previously stable course of the condition. The skin overlying the tender area did not show any specific lesions during the entire course of the illness. Examination revealed no lymphadenopathy or organomegaly.

## 3 METHODS

### 3.1 Differential diagnosis and investigation

**FIGURE 1:**
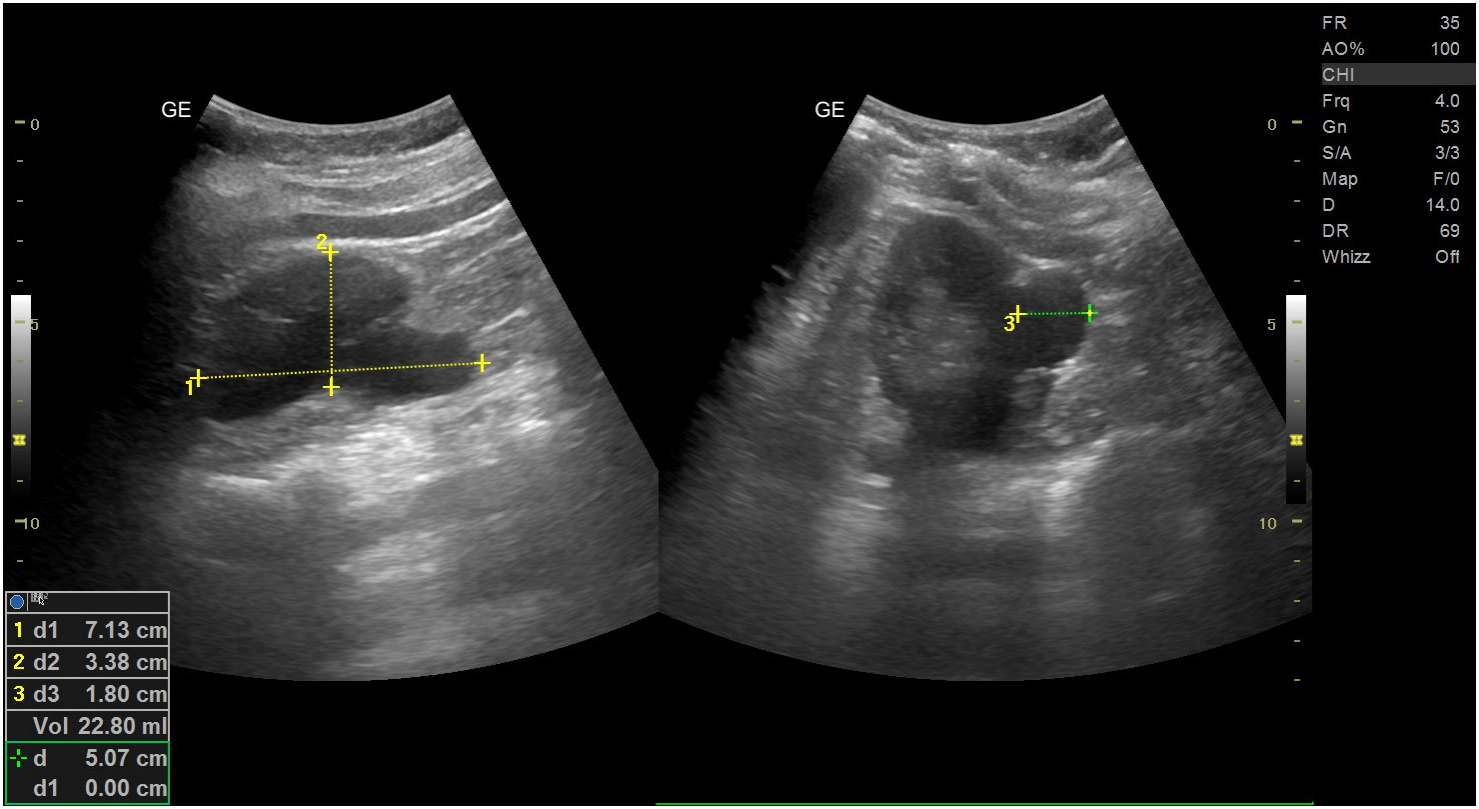
Ultrasonography representing left perinephric collection (abscess) with linear echogenic content.

The initial step in the diagnostic workup involved a complete blood count (CBC). Laboratory examination of blood revealed Hb of 12.6 g/dL and normal white blood cell count (total leukocyte count‐4000/cmm); differential count showed eosinophilia with 33% eosinophils, angiotensin‐converting enzyme‐2500/cmm, and erythrocyte sedimentation rate of 34 mm at the end of the first hour. Following the CBC, further investigation for unexplained fever was conducted. Tests for common infectious diseases endemic to the region were performed, including HIV, malaria, and tuberculosis. A rapid screening for HIV using the TRI‐DOT HIV test kit returned non‐reactive, and a subsequent fourth‐generation ELISA also returned negative. For malaria, a rapid diagnostic test (RDT) for Plasmodium antigens was negative. Additionally, a comprehensive sputum test for tuberculosis, including acid‐fast bacilli (AFB) staining and the GeneXpert MTB/RIF assay, yielded negative results. Widal test titers came within normal limits (Salmonella typhi “O” antibody titer <1:40, Salmonella typhi “H” antibody titer <1:40). IgM Anti HCV antibody and HBsAg (Hepatitis B surface Australian antigen) were not detected in the patient's serum by electrochemiluminescence immunoassay in Cobas 8000 auto‐analyzer (Roche Diagnostics). Lateral flow immunochromatography by Alere Filariasis Test Strip (Alere Scarborough) showed negative results for circulating filarial antigen. The daytime and nighttime peripheral blood smear examination did not reveal any microfilaria. With this clinical presentation, she was advised ultrasonography (USG) of the abdomen. Abdominal sonography was performed, revealing an unexpected finding—a left perinephric collection. This perinephric collection appeared anechoic on ultrasound (Figure 1), accompanied by the unusual observation of rhythmic movement of its contents referred to as the “filarial dance sign” (Data S1), suggestive of filariasis. The patient was recommended for a CT scan of the abdomen and pelvis to further investigate the perinephric collection, the patient was informed of its importance in confirming the diagnosis and ruling out other potential conditions. However, the patient declined the CT scan due to financial constraints. Therefore, the diagnosis was pursued with available clinical and sonographic findings. To further evaluate the possible presence of filariasis in other locations of the body, multiple commonly involved sites including lymph nodes, liver, lungs were checked using similar imaging technique, but nothing was found. Without any clinical examination, history, and imaging, revealing the possible involvement of other sites, it was confirmed to be an isolated case of left perinephric collection.

### 3.2 Treatment

Following the final diagnosis, treatment with albendazole and diethylcarbamazine (DEC) was prescribed for a period of 4 weeks.

## 4 CONCLUSION AND RESULTS

Upon follow‐up, the symptoms of fever and tenderness had subsided. Imaging was done once again; this time no filarial dance sign was observed. However, the lab reports still showed absolute eosinophilia. But the eosinophilia had reduced from 1080 to 650/mm^3^. Then radiological imaging of other body locations and peripheral blood smear test were performed one more time, again revealing no significant finding. Owing to the presence of eosinophilia, the drugs were continued for another period of 4 weeks. Upon the second follow‐up, the patient remained asymptomatic, eosinophilia had resolved, and lab reports were within normal ranges. As a consequence, the treatment was discontinued, and the patient was discharged.

## 5 DISCUSSION

Clinically, filariasis can be of two major categories—filariasis of skin and subcutaneous tissue and *lymphatic filariasis*. Onchocerca volvulus and *L. loa* are the most common organisms reported in the former, and *W. bancrofti* and *B. malayi* are the two most common species in the latter. *W. bancrofti* has been detected at different sites such as breast, thyroid, lymph node, liver, and lungs, and a small number of cases have been reported in the bone marrow and body fluids; however a perinephric abscess due to filariasis is extremely uncommon and has not been reported before in medical literature.^5^ Before observing the microfilaria on sonography, filariasis was not by far in the list of differential diagnoses, owing to the absence of the lymphatic involvement, unusual location, and negative blood smear test for microfilaria as the later still remains the main method of diagnosing filariasis. Diagnosing filariasis through blood smear tests requires consideration of periodicity, which refers to the specific time of day when microfilariae are most prevalent in the blood. This timing corresponds directly to the feeding habits of the various mosquito species responsible for transmitting the infection. Most mosquito species propagating lymphatic filariasis feed at night.^6^, ^7^ Therefore, blood samples for microfilariae detection are ideally collected during nighttime, preferably between 10 p.m. and 2 a.m. As the microfilaria of *W. bancrofti* often demonstrates periodicity, and the blood samples must be taken at night, preferably between 10 p.m. and 2 a.m. However, we conducted both day time and night time blood smear tests to diagnose and detect the causative organism, but both tests yielded negative results. A review of 18 cases of filariasis diagnosed on fine needle aspiration cytology (FNAC) revealed that only 2 (12%) were positive for microfilaria on blood examination.^8^ Due to the challenges associated with the low yield and stringent sampling requirements of a blood examination, alternative diagnostic approaches such as the preparation of buffy coat smear and FNAC can be considered for detecting the parasite, especially in asymptomatic cases. Additionally, in patients with atypical symptoms and eosinophilia in endemic areas, relevant imaging tests should be performed to identify isolated tissue involvement, as observed in this index case. In our case sonography was used to detect filarial dance sign after blood smear gave false negative results for infection, underscoring the utility of ultrasound in diagnosing filariasis even in atypical presentations. The “filaria dance sign” is a radiological finding observed during ultrasonography, typically indicative of filariasis. It refers to the characteristic, rhythmic, serpentine movement of adult filarial worms in the lymphatic vessels or tissue spaces.^3^ This sign is most commonly seen in cases of lymphatic filariasis, especially involving Wuchereria bancrofti. In ultrasound imaging, this movement creates a distinct pattern, suggestive of live worms. The sample from perinephric collection was not collected in our case for performing histological examinations, but W. bancrofti still remains the most suspected organism, owing to its high prevalence (98% of infections) in India. Therefore, index case may represent occult filariasis. It is a filarial infection in which microfilariae are not observed in the blood but may be found in other body fluids and/or tissues.^9^ Similar findings have also been reported by previous studies.^9^, ^10^ This highlights the issues associated with the most common diagnostic method for filariasis, indicating that other better and more reliable screening methods should be used in endemic countries where filariasis is prevalent and poses significant public health threat.

Our case can be confused with perinephric hematoma, perinephric abscess, or perinephric urinoma, as these conditions can clinically present with similar complaints and disease course.^11^ Thus, in patients with provisional diagnosis of these conditions specially in endemic tropical and subtropical countries such as India, a possible case of filariasis should be considered in the list of differential diagnosis. High index of suspicion is required to diagnose filariasis due to its wide range of clinical presentation and laboratory findings. It is also crucial to perform a comprehensive diagnostic workup that includes tests for common endemic infections like HIV, malaria, and tuberculosis. These diseases can present with clinical features similar to filariasis and are important differential diagnoses to consider.

In summary, our case presents an uncommon manifestation of filariasis with a distinct perinephric abscess. The diagnostic protocols exposed limitations in traditional filariasis detection methods, emphasizing the necessity for innovative screening approaches. While the administered albendazole and diethylcarbamazine effectively alleviated symptoms and reduced eosinophilia, the prolonged treatment duration prompts reflection on optimal and individually tailored therapeutic decisions. The atypical presentation underscores the challenges in filariasis diagnosis, urging a nuanced approach in endemic areas. This case contributes to the evolving understanding of filarial infections and underscores the imperative for ongoing research to refine diagnostic and therapeutic strategies in diverse clinical scenarios.

## AUTHOR CONTRIBUTIONS

**Govind Sharma:** Conceptualization; data curation; project administration; supervision; validation; visualization; writing – original draft; writing – review and editing. **Rajat Sachdeva:** Project administration; supervision; validation; visualization; writing – original draft; writing – review and editing. **Shweta Aggarwal:** Project administration; supervision; validation; visualization; writing – original draft; writing – review and editing. **Amogh Verma:** Validation; visualization; writing – original draft; writing – review and editing. **Ayush Anand:** Validation; visualization; writing – original draft; writing – review and editing. **Ajeet Singh:** Validation; visualization; writing – original draft; writing – review and editing. **Prakasini Satapathy:** Data curation; writing – review and editing.

## FUNDING INFORMATION

The authors did not receive any funding for this work.

## CONFLICT OF INTEREST STATEMENT

The authors have no conflict of interest to declare.

## ETHICS STATEMENT

Ethical approval was not required for this case report.

## CONSENT

A written informed consent was obtained from the patient based on the journal's policies.

## Supporting information


**Data S1.** Left perinephric collection demonstrating rhythmic movements of echogenic content “filarial dance sign”.

## Data Availability

Data sharing is not applicable to this article as no new data were created or analyzed in this study.
